# A longitudinal qualitative study of the UK general practice workforce experience of COVID-19

**DOI:** 10.1017/S1463423622000391

**Published:** 2022-08-04

**Authors:** Emily Burn, Rebecca Fisher, Louise Locock, Judith Smith

**Affiliations:** 1 Research Fellow at University of Birmingham, Health Services Management Centre (HSMC), Birmingham, UK; 2 Senior Policy Fellow, The Health Foundation, London, UK; 3 Professor in Health Services Research, Health Services Research Unit, University of Aberdeen, Aberdeen, UK; 4 Professor of Health Policy and Management, Director of the Health Services Management Centre (HSMC), University of Birmingham, Birmingham, UK

**Keywords:** COVID-19, patients, primary health care, qualitative research, remote consultation

## Abstract

**Background:**

The COronaVIrus Disease 2019 (COVID-19) pandemic has led to significant re-organisation of general practice in the United Kingdom and around the world. The general practice workforce has led changes to their services, often dealing with high levels of uncertainty. The way in which many practitioners consult has shifted significantly, and there has been an increase in the number of phone and online consultations. We know very little about how those working in general practice experienced the service reorganisation introduced in the first year of the COVID-19 pandemic.

**Aim:**

The aim of this project was to describe the changes in the delivery of general practice in the United Kingdom in the first year of the COVID-19 pandemic. Furthermore, to explore primary care practitioners’ and managers’ experiences of change within general practice during this time and investigate shifts in perceptions of professional identities.

**Method:**

We conducted a longitudinal qualitative study that captured narrative accounts from 17 primary care practitioners and managers across England and Scotland. Each participant submitted narrative accounts in the first year of the COVID-19 pandemic using self-recorded or written contributions, or via an interview if preferred. These were analysed using a grounded theory approach, with thematic coding used to construct common themes.

**Findings:**

Participants’ narratives describe the challenges COVID-19 presented to general practice. Responses mirror the shifts in the pandemic and its management – from an initial sense of autonomy but uncertainty, to a period of stability and patients’ increasing frustration. The re-organisation of general practice has affected practitioners’ views of their work and their role as clinicians. Participants’ narratives were framed profoundly by the importance of their relationships with patients. This analysis of practitioners’ and managers’ narratives highlights the need for further exploration of how to support the general practice workforce’s well-being longer term in a context of increased demand and significant change.

## Introduction

The COVID-19 (COronaVIrus Disease 2019) pandemic has necessitated significant changes in general practice in the United Kingdom (UK). The need for infection control precautions precipitated a sharp rise in the number of telephone and video consultations and a fall in face-to-face consulting (NHS Digital, 2021). Between April and June 2020, general practitioners were limited to making only urgent referrals to secondary care. Simultaneously, general practitioners had to get to grips with the clinical management of a novel virus. This included service re-design, such as the establishment of ‘hot hubs’ to treat patients with COVID-19 symptoms. COVID-19 required general practice to alter radically the way it operates, quickly instigating changes that might previously have taken years to embed (Brant *et al*., 2016; Marshall *et al*., 2020; Greenhalgh and Rosen, 2021).

The tumult generated by COVID-19 also created a sense of opportunity to innovate and change a model of general practice viewed by many in the UK to require improvement (Rosen, 2015; Baird *et al.*, 2016). Healthcare systems around the world introduced different changes to primary care services in response to the COVID-19 pandemic (Haldane *et al*., 2020; Huston *et al*., 2020). However, the UK was not unique in experiencing a shift from face-to-face to remote consulting (Patel *et al*., 2021a; 2021b; Alexander *et al*., 2020).

Previous studies have analysed the shifts in general practice activity throughout the COVID-19 pandemic (Sharma *et al*., 2020; Watt *et al*., 2020; Murphy *et al*., 2021; Clarke *et al*., 2022), but none appear to have examined the lived experience of the general practice workforce throughout this period of change. We address this gap through a longitudinal series of narrative accounts from the general practice workforce, reporting the changes made to service delivery and their experiences of living and working through COVID-19 in real time.

The UK has experienced rolling waves of COVID-19. In the period covered by this study, the ‘first wave’ of COVID-19 – which peaked in late March/early April 2020 – was followed by a second wave from September 2020 which peaked in early-January 2021 (Office for National Statistics, 2021). The rollout of the vaccination programme in the UK from December 2020 has been noted to reduce hospitalisations and deaths from COVID-19 (Public Health England, 2021). This paper seeks to explore the general practice workforce’s experiences of the shifting pandemic and changes to service delivery made in response to the first year of COVID-19.

## Method

The research team has an interest in workforce issues in general practice and were keen to explore the impact of COVID-19 on practitioners’ and managers’ professional identities. From April 2020, narratives were collected from general practitioners, practice nurses, and practice managers, with the last submission received in March 2021.

A purposive sampling approach (Blaikie, 2009) was used to recruit participants to ensure a mixed sample that represented different geographical locations and different levels of experience. Potential participants identified by the research team were sent an introductory email. A small number of participants were known in a professional capacity by one or more members of the research team.

We targeted all regions across England and aimed to include participants based in Scotland and Wales. Within our sample, six participants were based in the West Midlands, five in the southeast of England, four in London, and two in Scotland. While we did not secure participants from all regions of England, or from Wales, our sample encompassed a range of levels of experience and both partnered and salaried general practitioners. Salaried general practitioners are employed by their practice, whereas general practice partners are self-employed, generally having greater influence over the direction of the practice, and sharing decision-making responsibility with other partners in the practice.

Participants were informed that the study aimed to capture the experiences of practitioners and managers at a time of great change in general practice. Three potential participants expressed interest in the study but did not progress to submit a narrative. The research team used a grounded theory approach where theory and concepts are generated from the data (Glasner and Strauss, 1967). The research team devised and regularly revised open questions which reflected recent developments within the pandemic and its management. Participants were informed that they should not feel bound by these questions and were free to discuss the most pressing issues of concern and divulge personal accounts of their experiences. Furthermore, participants were free to contribute to the study as frequently as they wished. However, the open approach to data collection meant that the number of submissions received across the participant group varied.

In total, batches of questions were sent to participants at six points throughout the first year of the pandemic. There was a range of options for participants to share their narratives. Some preferred to record voice notes on their phone that were shared securely with the research team. Others submitted written notes or were interviewed via telephone or an online platform. Interviews were completed by non-clinical researchers (EB and LL) to ensure that discussions did not fall back on assumed shared understandings (Chew-Graham *et al*., 2002), and this approach aimed to generate a greater depth of data.

All interviews were audio recorded. Interviews and voice notes were professionally transcribed, loaded onto NVivo version 12 (QSR International, 2018), and then coded by one member of the research team (EB). Thematic coding was used to construct key topics within the data (Braun *et al.*, 2018) throughout the data collection. The data were first coded using the suggested open-ended questions posed to participants to structure their responses (Table 1).

**Table 1.** Example of open-ended questions sent to participants to structure responses

What changes have been made in your practice in recent weeks? What has been difficult in recent weeks? What has been positive (if anything) about recent changes? Have you been involved in the COVID vaccination programme? If so, please tell us about your experience, including what you’ve been doing, what has/hasn’t been going well and how you’re feeling about it? Do you have any thoughts on how the pandemic might affect your practice and your profession in future? How is the COVID-19 pandemic affecting you personally in the context of the rest of your life? Is there anything else you’d like to add, or anything that has been on your mind about primary care and COVID recently?

Following data collection, the research team produced an overview of the themes within the data. Analysis explored chronological elements within each theme and findings are presented thematically, from the start of the pandemic to the establishment of the vaccination programme.

Frequent research team meetings throughout the study period served to develop the coding frame and revise the questions put to participants as the pandemic evolved. Additional questions addressed, for example, guidance for clinically extremely vulnerable people, participants’ experience of the vaccination programme, and how participants’ practices were preparing to ‘co-exist’ with COVID-19. Multiple iterations of coding took place and generated further key themes in the data. The research team considered a selection of coding reports within these sessions and discussed emerging findings. One member of the team (RF) is a general practitioner. This expertise informed data analysis and offered the opportunity to test and develop initial interpretations.

### Findings

Seventeen participants submitted narratives across the study (13 general practitioners, two practice nurses, and two practice managers). Of the general practitioners, nine were partners and four were salaried general practitioners. In total, 55 submissions were received from participants, with an average 3.2 submissions being contributed per participant. There was some degree of drop-off from participants as the project progressed which could be expected given the demands placed on participants’ time and the open data collection methods used within the project.

### Initial change: ‘Almost like a tsunami passing through your life’

At the initial stages of the pandemic, participants spoke about the huge changes made to the organisation and delivery of general practice services. Many participants spoke of the difficulty of keeping up with constantly changing guidelines on the management of COVID-19 patients and the delivery of services for non-COVID-19 patients:

‘There is a massive increase in guidelines/protocols and URGENT MUST-READ documents’ Participant 3

‘I could write a book on the changes we tried and abandoned or tried and then evolved and it’s vital this is captured for the future but to put it in perspective, after eight weeks (since approx. 10 March) we have had 21 versions of what we now call our practice Standard Operating Procedures for Clinical Encounters… At one stage it was changing daily, we are now trying to limit it to once a week’. Participant 5

‘I think another thing that’s been challenging is the volume of emails and updates that we’re getting… And so you find yourself constantly trying to dig out emails when you’re speaking to patients, trying to work out what the most up to date way of managing the situation is, and that all just adds time to appointments’. Participant 1

Some participants noted that the pandemic had sped up the implementation of positive changes:

‘…so the Health Boards told practices that they had to look after – or each care home and each nursing home now has only one practice looking after its residents, which is something that was just so obvious to do… it’s the right thing to do in so many ways – but the practices in [PLACE] could never get themselves organised to do that… Now they’ve been forced to do that and that’s such a good thing’. Participant 2

Participants often described an initial sense of liberation and freedom, as the usual bureaucracy was swept away to allow service changes to be made quickly.

‘So it was just a huge change really and it was no good kind of waiting for national guidance, from NHS England really, about what to do. I think it really was individual practices and maybe practices speaking within their Primary Care Networks, to sort of come up with a plan really and think how to work it’. Participant 12

For example, general practitioner appraisals and routine inspections by the Care Quality Commission (the independent regulator of health and adult social care services in England) were suspended in the early phase of the pandemic. Participants referred to a newfound openness of the system for ideas to be tested out and adapted:

‘And I think what the pandemic really played into is that, sort of, the natural agility that you see in general practice… So everyone was so burnt out, a bit broken by the old way of doing things that, you know, such a radical change comes along it, it just felt the right thing to do and that was quite exciting in a weird way… and there’s too many yardsticks that we’re measured by that change every year to define what we feel as good quality care and I think it was just, it was really nice to feel that actually we could look at a clinical problem and say this is, this is what we have available. This is what we think we can do. We’re going to do it and just kind of get on and do it’. Participant 11

However, the perceived increased autonomy within practices was also met by feelings of great uncertainty from some participants as ways of working and modes of consulting changed.

### A period of stability and gradual restoration: ‘But you know, you’ve got to take a little bit more risk in a way’

Over time, participants reported that the level of initial change had settled down, however, for some, this was accompanied by a creeping return of bureaucracy and a reduced sense of autonomy for practices and their staff. Nevertheless, in the main, participants welcomed a period of stability.

Throughout this period of stability, participants acknowledged often that general practice had changed and, in the words of one participant, ‘*a lot of it won’t go back to normal’* (participant 7). Participants reflected frequently that there was an underlying uncertainty as to what the future of general practice would look like and that they and colleagues were dealing with great weariness and unease.

Some general practitioner participants reflected that the introduction of remote working had made it easier to schedule multidisciplinary team meetings and had reduced the amount of time spent travelling to meetings and events. However, others reflected that the boundaries between their home and professional life had become more blurred with the perception that you are ‘*on call the whole time*’ (participant 11).

### Patients’ responses to the COVID-19 pandemic: ‘And people, you can tell, are getting more fed up and grumpy’

The majority of practitioners’ discussions of general practice during COVID-19 were interpreted through their relationship with patients. In the very initial stages of the pandemic, practitioner participants reported that patients tended to avoid contacting the practice and ‘*very much followed the government advice of don’t overwhelm the NHS’* (participant 4). However, as the pandemic progressed, there was an increased demand for consultations as pent-up demand was revealed, people felt more confident to leave their home, and with more face-to-face appointments taking place. Some also reported a by-product of remote consulting was the potential to create ‘*supply-induced demand’* (participant 1), and participants did report growing demand for consultations during the winter of 2020–2021. As the pandemic progressed, participants reported a growing workload and increasing pressure and stress as a result.

‘We had a bit of an explosion because we’re getting lots and lots of contacts because people find it so easy now, I suppose’. Participant 13

Practitioner participants often identified with general practice’s strong roots within the community. This emphasis on the local community was evident when participants spoke about the positive relationship patients had with the practice and the value of continuity of care. As the pandemic progressed, and the pressures of social distancing became more acute, participants noted that the relationship between practice and patient could become strained at times. Some participants noted an increasing tendency for patients to perceive that the practice was ‘closed’ and discussed negative portrayals of general practice in the media, noting *‘that everywhere you look, in [the] media over the past few months, whatever paper you read, there’s GP [general practitioner] bashing going on’* (participant 17). As another participant noted:

‘It doesn’t help with the media I think, you know, saying GPs are closed and actually probably fuelling people’s anxiety about how they access GPs’. Participant 12

Participants often spoke of the increasing sense of anger from some patients who felt they were not being adequately served by their general practice, with reception staff often bearing the brunt of this frustration:

‘You know, so we went through a point where everyone was fairly happy that we’d been doing a great job, and then overnight people being like ‘oh, so I can speak to you, you’re open’. I’m like, ‘I’ve spoken to you six times this year! Of course we’re open! I saw you last week’’. Participant 11

### Relationship with role as a primary care practitioner: ‘I feel like my clinical sparkle has been dulled’

A number of participants reflected on the way in which COVID-19 had affected their perceptions of their role as a general practitioner. Remote consultations were described by some participants as feeling transactional and in opposition to the relationship-based care of general practice which they valued highly as a core feature of general practitioner and practice nurse roles. Practitioner participants noted that they could no longer rely on the cognitive shortcuts developed through their experience of consulting face-to-face, for instance the patient’s gait as they enter the consulting room. Initially, participants also expressed some concern about how the move to remote consulting might affect their ability to build and sustain rapport with patients. However, the majority reported that they had quickly become accustomed to remote consultations and noted that a higher proportion occurred via telephone than by video consultation. Some discussed that remote consultations seemed to benefit patients who appeared to feel more comfortable talking over the telephone rather than discussing their concerns face-to-face. Nevertheless, a small number of general practitioner participants discussed experiencing a sense of loss and that they were missing the more profoundly relational aspect of general practice. Some attributed this to missing routine face-to-face contact with patients, in addition to weariness with responding to COVID-19 and the efforts being made on a daily basis to control the spread of the virus.

General practitioners were not immune to the wider consequences of the pandemic on their personal and professional lives. One participant discussed that they were feeling flat and found that their day-to-day work was not as fulfilling as it was prior to the pandemic (participant 5). This participant reflected that at the initial stage of the pandemic, there was a sense of being in ‘*crisis reaction mode*’ (participant 5) which then receded as the level of infections decreased. As the following quotation demonstrates, the participant reflects that during the pandemic, they felt they held more risk on behalf of patients and were more concerned about those in their care. The additional mental load was reflected to have taken its toll on how the participant perceived their role as a general practitioner:

‘And I’m not depressed and I’m not angry, I’m just sadder. And I come home from work and I know I’ve done the best job I can, but I’m often more worried about my patients on average than I used to be, because I haven’t satisfied my professional enquiry that I’ve done – have I ruled everything out? Have I missed anything, you know? And that just takes its toll over time…’. Participant 5

A number of participants reflected on how the pandemic had influenced their perceptions of their role as a general practitioner, as captured in this below quotation:

‘I’m slightly frightened, that that’s going to be difficult… I won’t work unless I can be the GP I want to be, I’m not a sort of, and I know what that looks like, but to be fair, I’m pretty sure that it’s what my patients would want anyway. It’s just that kind of moment of encounter to be, you know, authentic and meaningful to the patient’. Participant 10

Here, the changes to general practice instigated by COVID-19 are acknowledged to have potential long-term effects on how the participant relates to their role.

### The COVID-19 vaccination programme: ‘A real sense of satisfaction’

The rollout of the COVID-19 vaccination programme from December 2020 was strongly supported by study participants. While some initial teething problems were discussed, participants were positive about both the logistics of the rollout, as well as the implications of the vaccination programme for their patients.

Towards the close of 2020, participants often spoke of a sense of fractured relationships with some patients. It is notable that the vaccination programme offered the occasion to restore and renew these relationships and emphasise the central and important role of general practice within the local community:

‘…it feels like a hopeful, positive thing to do. As GPs and a primary care team who work really closely with a community, I think it feels like a lovely thing to do with, and for, that community and I think in policy terms it makes a lot of sense for general practice to be involved in delivering the vaccine.’ Participant 1

## Discussion

The intense demands that the first year of COVID-19 presented for general practice in the UK are reflected in this study. Participants repeatedly emphasised the significance of their relationships with patients and the importance of feeling that they are delivering high-quality care. With general practice in the midst of a nursing and medical workforce crisis, the shifts in professional identity noted in this study and precipitated by the pandemic must be further explored and understood. The nation can ill afford an exodus of general practitioners and practice nurses.

General practice across the UK was under resourced before the COVID-19 pandemic (Beech *et al.*, 2019), and consultation numbers are now consistently above pre-pandemic levels (NHS Digital, 2021). Our participants spoke of their concerns about managing an increasing workload within general practice and whether the advancement of remote consulting might further increase unsustainable demands.

This concern appears to echo the modelling of Salisbury *et al.* (2020) who suggest that digital-first approaches are likely to lead to increased workload for general practitioners unless consultations are shorter, or fewer patients require subsequent face-to-face consultations. However, Salisbury *et al.* (2020) note that there is as yet little available evidence on whether remote consultations increase patient demand.

As well as affecting workload, some general practitioner participants discussed their uncertainty about the future delivery of general practice and how they will interpret and relate to their role. These participants questioned whether they would derive sufficient job satisfaction in a world of remote-first consulting. Elsewhere (Burn *et al*., 2021), we explore the effects of the COVID-19 pandemic on participants’ sense of professional identity and call on Bury’s (1982) concept of biographical disruption and Ashforth’s (2000) transition bridges to explore how participants navigated these changes.

Previous research has found that practitioners have experienced high levels of stress and fatigue during the COVID-19 pandemic (Xu *et al*., 2020; Trivedi *et al*., 2020; Di Monte *et al*., 2020; Sharma *et al*., 2020; Sotomayor-Castillo *et al*., 2021). However, the majority of these studies are based on cross-sectional quantitative research designs. The longitudinal design employed by this study demonstrated how relationships between patients and their general practice had been challenged. Furthermore, previous studies have found that the advancement of remote consulting within general practice has broad support from practitioners (Donaghy *et al*., 2019; Murphy *et al*., 2021). However, research has indicated that adequate infrastructure needs to be in place to optimise remote consultating (Sharma *et al.*, 2020; Wherton *et al.*, 2020; Greenhalgh *et al*., 2021; Murphy *et al*., 2021). Remote consultations have been proposed to be more appropriate for ‘transactional presentations but are of uncertain and untested value for relational ones’ (Marshall *et al*., 2020, p. 270). By way of contrast, our participants did note that they could form positive relationships with patients when consulting remotely. However, as also found by Murphy *et al*. (2021), participants noted that they were holding higher levels of risk, with more complex presentations coming forward as the pandemic progressed.

Findings from previous studies have suggested that remote consultations may increase workload in the long term (Salisbury *et al*., 2020; Murphy *et al*., 2021; Turner *et al*., 2022). Our findings suggest that primary care practitioners are experiencing high levels of weariness and an ever-growing workload, a finding supported by NHS Digital data and surveys of UK general practitioners (Fisher *et al*., 2020; NHS Digital, 2021). Furthermore, the economic and social ramifications of COVID-19 will have long-term effects and general practice will be the first port of call for many people (Gray and Sanders, 2020). It remains to be seen how primary care services will meet this demand.

Our study demonstrates the urgent need to build on the findings of previous studies and explore how the primary care workforce is responding to the new ways of working brought about by remote-first consulting. This exploration should consider what would support primary care practitioners in their work and wider well-being and ensure sustainability of the general practice and primary care nursing workforce in the face of increasing demand for services.

### Strengths and limitations of the study

The longitudinal study design captured the shifting currents of the first year of the COVID-19 pandemic. Data were collected from the early stages of the pandemic and captured the dynamics of the introduction and subsequent easing of lockdowns on the experiences of primacy care practitioners and managers. The study’s focus on how participants perceive and relate to their role allows us to draw out potential ramifications of the pandemic on the future delivery of primary care.

Findings from this study reflect the in-depth experience of 17 general practice staff recorded over time. While the sample is relatively small, our findings nonetheless have wider relevance for the NHS general practice workforce. Participants were asked to reflect on the effects of COVID-19 on their wider home life, if they were comfortable to do so. To the authors’ knowledge, there are few longitudinal qualitative or mixed methods studies focusing on the personal experience of being a primary care practitioner during a time of COVID-19. Murphy *et al*.’s (2021) study on experiences of remote consulting is an exception. The focus on tracing the trajectory of COVID-19 in general practice captures the shifts in the pandemic and the effects on service delivery. This study therefore presents a comprehensive account of practitioners’ and managers’ personal experience of the COVID-19 pandemic within general practice.

The study attempted to collect narratives from general practice nurses and practice managers to ensure the representation of a wide variety of experiences within general practice. However, despite our attempts to recruit a mixed sample, most participants were general practitioners. Consequently, we were unable to undertake separate analysis of the experiences of these different participant groups. Furthermore, our participants were predominantly based in England. Recruiting a greater mix of participants would allow for further comparison of experiences across the four nations of the UK.

### Implications for research and/or practice

The UK general practice workforce was already overstretched at the onset of the COVID-19 pandemic and continues to be under a great deal of strain. Demand for appointments has increased at the same time as the number of full-time equivalent general practitioners has declined and there are long-term staff shortages (NHS Digital, 2021; The King’s Fund, 2021). Attempts have been made to recruit and retain the general practice workforce in the UK; however, the number of general practitioners remains comparable largely due to increased part-time working (House of Commons Library, 2018; NHS England, 2021; Review Body on Doctors and Dentists’ Remuneration, 2021). Remote consultations have become more frequent during the COVID-19 pandemic. Nevertheless, our understanding of the impact of increased remote consulting on practitioner and patient expectations and experiences, access, and quality is still emerging (Salisbury *et al*., 2020; Greenhalgh and Rosen, 2021; Ray and Mash, 2021). This study contributes to our appreciation of practitioners’ and managers’ personal experience of the first year of the COVID-19 pandemic and the shifts brought about within their professional identity.

The COVID-19 pandemic is unparalleled, and the long-term effects on primary care and the general practice profession are as yet unknown. The sustainability of general practice is dependent on ensuring there is an adequate supply of practitioners, and foregrounding practitioner well-being may support the retention of the general practice workforce.

At the initial stage of the pandemic, when practices were thrust into developing a response to COVID-19, participants spoke of the sense of almost liberation and increased autonomy for practices and this initial increased autonomy appeared to re-energise some participants in our research. Investigating how an increased sense of autonomy could be embedded within training and regulatory frameworks may offer a further avenue to explore how to address burnout and exhaustion within general practice. The additional pressures of the COVID-19 pandemic draw attention to the need to find new and more effective ways in which to support the workforce in order to ensure the sustainability of general practice that has changed in profound ways and will likely never be the same again.
